# HD-EEG for tracking sub-second brain dynamics during cognitive tasks

**DOI:** 10.1038/s41597-021-00821-1

**Published:** 2021-01-27

**Authors:** A. Mheich, O. Dufor, S. Yassine, A. Kabbara, A. Biraben, F. Wendling, M. Hassan

**Affiliations:** 1 Neurokyma, 35700 Rennes, France; 2 L@bISEN-Yncréa Ouest, ISEN, Brest, France; 3 Univ Rennes, LTSI - U1099, F-35000 Rennes, France; 4 Neurology department, CHU, Rennes, 35000 France

**Keywords:** Electroencephalography - EEG, Cognitive neuroscience

## Abstract

This work provides the community with high-density Electroencephalography (HD-EEG, 256 channels) datasets collected during task-free and task-related paradigms. It includes forty-three healthy participants performing visual naming and spelling tasks, visual and auditory naming tasks and a visual working memory task in addition to resting state. The HD-EEG data are furnished in the Brain Imaging Data Structure (BIDS) format. These datasets can be used to (i) track brain networks dynamics and their rapid reconfigurations at sub-second time scale in different conditions, (naming/spelling/rest) and modalities, (auditory/visual) and compare them to each other, (ii) validate several parameters involved in the methods used to estimate cortical brain networks through scalp EEG, such as the open question of optimal number of channels and number of regions of interest and (iii) allow the reproducibility of results obtained so far using HD-EEG. We hope that delivering these datasets will lead to the development of new methods that can be used to estimate brain cortical networks and to better understand the general functioning of the brain during rest and task. Data are freely available from https://openneuro.org.

## Abstract (summary)

**Table:** 

Measurement(s)	brain measurement • cognitive behavior trait
Technology Type(s)	electroencephalography (EEG)
Factor Type(s)	task • age • sex
Sample Characteristic - Organism	Homo sapiens

Machine-accessible metadata file describing the reported data: 10.6084/m9.figshare.13560311

## Background & Summary

Emerging evidence shows that brain (dys)functions arise from communications between spatially distant brain regions^1,2^. Although functional MRI has revolutionized neuroscience in the last decades, its intrinsic poor time resolution (>sec) is a major drawback limiting its use in tracking fast brain network dynamics that underlies the execution of several brain (cognitive and perceptivo-motor) processes. Electro/Magneto-encephalography (EEG/MEG) are unique non-invasive techniques, which enable the tracking of brain dynamics on a millisecond time-scale. Several studies have been done to track cortical brain networks, using the EEG/MEG source connectivity method, during task-free^3,4^ or task-related paradigms^5^. However, despite laudable efforts on the model of MEG dataset from Human Connectome Project (HCP)^6,7^ and several EEG datasets^8,9^, only few are available for both rest and task and open-access high density EEG (HD-EEG, 256 channels) data during different task are still missing.

The HD-EEG combined with sophisticated signal processing algorithms is increasingly transforming EEG into a potential neuroimaging modality^10,11^. Recent EEG studies revealed the possibility to track fast dynamics of functional connectivity at rest^12^ and during cognitive tasks^13,14^. Also, some studies reported the potential use of HD-EEG data (compared to low EEG channel density) in some pathological conditions such as the localization of epileptic networks^15^ and the detection of cognitive decline in neurodegenerative diseases^16^. In addition, emerging evidence show the possibility, to some extent, to capture sub-cortical structures using HD-EEG^17,18^. In this context, the availability of task-free and task-related open-access HD-EEG databases is becoming mandatory to (i) decipher the fast (sub-seconds) reconfiguration of functional brain networks during cognition, (ii) develop new signal processing methods to adequately estimate cortical brain networks and (iii) allow the reproducibility of results obtained so far using HD-EEG.

Here, we provide the first open-access HD-EEG (256 channels) datasets recorded during resting state and four different tasks (visual naming, auditory naming, visual spelling and working memory). Parts of the data have already been used for developing and analyzing various signal processing methods. In particular, our efforts were focused on the estimation of functional brain networks at rest and during picture naming^12,14,19–22^. None of these studies, however, have described the database in details, and previous works have so far only used small parts of the data presented. In this work, we provide all the necessary details and an open-access to the database so that the international scientific community can freely generate greater understanding of brain functions during task-free and task-related paradigms. This will also ease the development of new methods to improve the accuracy of currently used techniques to estimate cortical brain networks using HD-EEG and to confront these techniques each other by comparing results and to run future meta-analyses. We hope that this dataset will help (among other objectives) to make EEG source-space network analysis a mature technique to address some of the questions in cognitive and clinical neuroscience.

## Methods

### Data collection

**Fig. 1:**
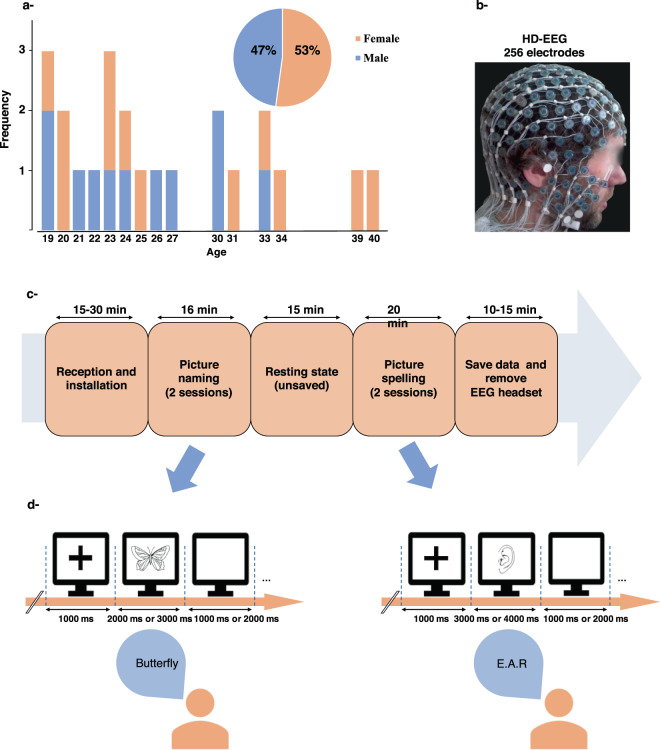
Dataset 1, (**a**) Participants (N = 23). (**b**) HD-EEG system used in the experiment. (**c**) A representative schema of the dataset 1 collection procedure. (**d**) Experimental design for pictures naming and spelling.

**Fig. 2:**
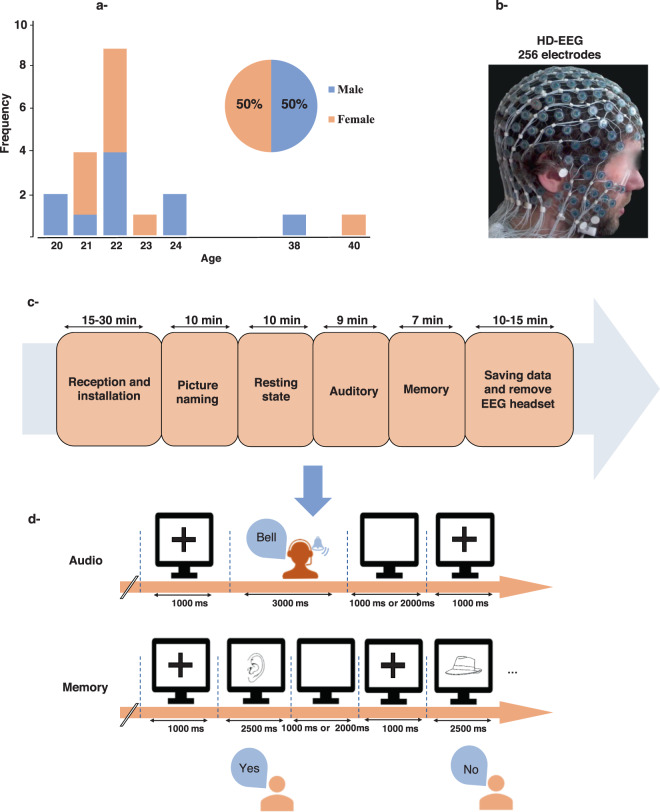
Dataset 2. (**a**) Participants (N = 20), (**b**) HD-EEG, (**c**) Four tasks (visual naming, auditory naming, memory task and resting state). (**d**) Experimental design for auditory and memory tasks.

Data were collected between 2012 and 2017 in Rennes (France) during two different experiments. The first dataset consists of naming and spelling the names of visually presented objects (Fig. 1). The second dataset includes resting state, visual/auditory naming and visual working memory tasks (Fig. 2). The same equipment was used in both datasets and recordings were performed in the same place (Rennes University Hospital Center). HD-EEG system (EGI, Electrical Geodesic Inc., 256 electrodes) was used to record brain activity with a sampling rate equal to 1 KHz and electrodes impedances were kept below 50 kΩ. Involved participants were different for the two studies. They provided their written informed consent to participate and fulfilled some inclusion/exclusion criteria questionnaires (summarized in Table 1). The participants were seated in a medical armchair linked to the faraday structure of the room. The room was lit by natural light attenuated by blinds. Heads of our participants were approximately located 1 m in front of the 17′screen. Images were presented centrally as black drawings on a white background without any size modification (10 cm × 10 cm). This setup corresponds to a viewing angle of 2.86 degrees of maximum excenticity from fixation point so that the entire image falls within the participant’s foveal vision. Sounds were displayed through 50 watts logitech speakers without any possibility of audio isolation.

**Table 1:** Inclusion and exclusion criteria. .

Inclusion criteria	Exclusion criteria
>18 years	Psychiatric/neuropsychological/developmental pathologies
	Bilingualism or multilingualism
Right-handed	Visual disorder
French native speakers	Delay in learning French (oral/written)
Written informed consent	Taking medication/treatment, others that can affect brain functioning
	Pregnant/breastfeeding women
	Persons under guardianship, curatorship or protection of a conservator

#### Dataset 1

Participants. Twenty-three right-handed healthy volunteers of whom 12 females, with an age range between 19–40 years (mean age 28 year), and 11 males with an age range between 19–33 years (mean age 23 years) participated. This experiment was approved by an independent ethics committee and authorized by the French institutional review board (IRB): “Comité Consultatif de Protection des Personnes dans la Recherche Biomédicale Ouest V” (CCPPRB-Ouest V). This study was registered under the name “conneXion” and the agreement number: 2012- A01227-36. Its promoter was the Rennes University Hospital.

Experimental procedure and design. The experiment begins with the verification of inclusion/exclusion criteria. The participants read the information notice and the consent form. Then, those who signed to participate completed two questionnaires. The first questionnaire collects information related to the inclusion/exclusion criteria and to personal information (name, age, sex, address), while the second one allows to determine the manual laterality of the participants using the Edinburgh manual laterality measurement scale adapted in French (Edinburgh Handedness Inventory, Oldfield, 1971^23^). Afterwards, the acquisition procedure is explained to the participant (see Fig. 1c). The experimental paradigm includes two conditions, the first one corresponds to the picture naming task and the second one to the picture spelling task. The spelling task always follow the naming task and its instruction was not given before the naming task was completed to avoid any reminiscence of words orthographic structures

Both naming and spelling tasks are divided into two runs of 74 stimuli to avoid tiredness of participants. Each run contains balanced numbers of animals and objects as well as long and short words. Pictures are presented on a screen using a computer and the experimental paradigm is diffused using E-prime® Psychology Software Tools ©^24^. The responses produced by the participants are collected via a Logitech® microphone and analyzed to detect onsets of speech using Praat v5.3.13 (University of Amsterdam, 1012VT Amsterdam, The Netherlands)^25^.

***Task 1: Picture naming***. Participants were asked to name at a normal speed 148 displayed pictures on a screen divided in two runs of about 8 min each. The images were selected from a database of 400 pictures standardized for French^26^. The presented pictures represent two categories (animals (74) and tools (74)). Naming agreement of picture is very high leading to few mistakes while recovering words associated to images. The word length is controlled such that the paradigm includes 74 short words (37 animals and 37 tools) of 3 to 5 letters and 74 long words (37 animals and 37 tools) of 7 to 10 letters. Other psycholinguistic parameters were controlled to get equivalent datasets (name agreement, image agreement, age of acquisition as well as linguistic parameters like oral frequency, written frequency, letters/phonemes/syllables and morphemes numbers), see Table 2.

**Table 2:** Psycholinguistic parameters controlled to get equivalent datasets for each category of pictures in dataset 1 (Animals vs Tools).

	AVERAGE Animals	AVERAGE Objects	STDEV Animals	STDEV Objects	T-test Animals vs Objects
Name agreement (%)	**94,3243**	**95,8378**	7,5058	5,8572	0,3370
Image agreement (average)	**3,7550**	**3,6126**	0,4481	0,7356	0,3189
Age of acquisition (average)	**2,3292**	**2,5289**	0,6152	0,5819	0,1557
Number of letters	**5,9730**	**6,1622**	1,9506	1,9792	0,6800
Number of phonemes	**4,2162**	**4,2703**	1,7502	1,6608	0,8920
Number of syllables	**1,7297**	**1,7027**	0,8383	0,7769	0,8860
Number of morphemes	**1,0541**	**1,1351**	0,2292	0,4191	0,3064
Oral frequency	**12,1324**	**9,1903**	13,9555	6,9085	0,2556
Written frequency	**11,1673**	**11,0351**	11,3952	6,8723	0,9520

All pictures were shown as black drawings on a white background. The order of presentation within a run of 74 stimuli was fully randomized across participants. Naming latencies were determined as the time between picture onset and the beginning of vocalization recorded by the system. EEG Triggers of images that were not correctly recognized or not recognized at all are discarded so that they don’t appear in the dataset.

***Task 2: Picture spelling***. In this task, participants were asked to spell the same images used in the picture naming task. The instruction about the spelling task was given after the completion of the naming task to keep the participant naïve about our interest to the spelling. Also, this task always happened after the picture naming task to avoid the re-activation of the spelling of image’s names. Two sessions of visual spelling were performed for each participant and the duration of each session was about 9 to 10 *min*. This task is very similar to the previous one except that it involves the orthography of words that correspond to the named images. One can easily recognize the drawing and named it without going through activation of the orthography of the word. In spelling, recovering the exact orthographic structure with the sequence of letters is an additional step that closes spelling and writing. This task includes the same 148 images selected from the database of 400 pictures standardized for French^25^ that were used for the naming task.

These data have not been extensively analyzed, especially under the psycholinguistic angle. They have also never been compared together with the picture naming task.

#### Dataset 2

Participants. Twenty right-handed healthy volunteers (10 females, 10 males, mean age 23 years- See Fig. 2a) participated in this experiment. Like for the dataset 1, all participants provided a written informed consent to participate in this study which was approved by an independent ethics committee and authorized by the IRB (CCPPRB-Ouest V)). Recorded study name was “Braingraph” and the study agreement number was 2014-A01461-46. Its promoter was still the Rennes University Hospital.

Experimental procedure and design. The experiment is composed of four tasks: resting state, picture naming, auditory naming and working memory. The three first tasks are distributed in a counterbalanced way within the group. The memory task always happened at the end of the session because it involved 40 images from the previous naming task amongst its 80 displayed images. All responses for each trial within the tasks are available with the data. Triggers corresponding to false responses or no responses are simply discarded from the dataset.

***Resting state EEG***. The participants were asked to relax for 10 minutes with their eyes opened during the recordings. Participants were facing the computer screen which displayed a fixation cross. They were told not to fixate the cross but to keep their eyes in the vicinity of it so that the rest run could also be used as a control for visual or auditory naming.

**Fig. 3:**
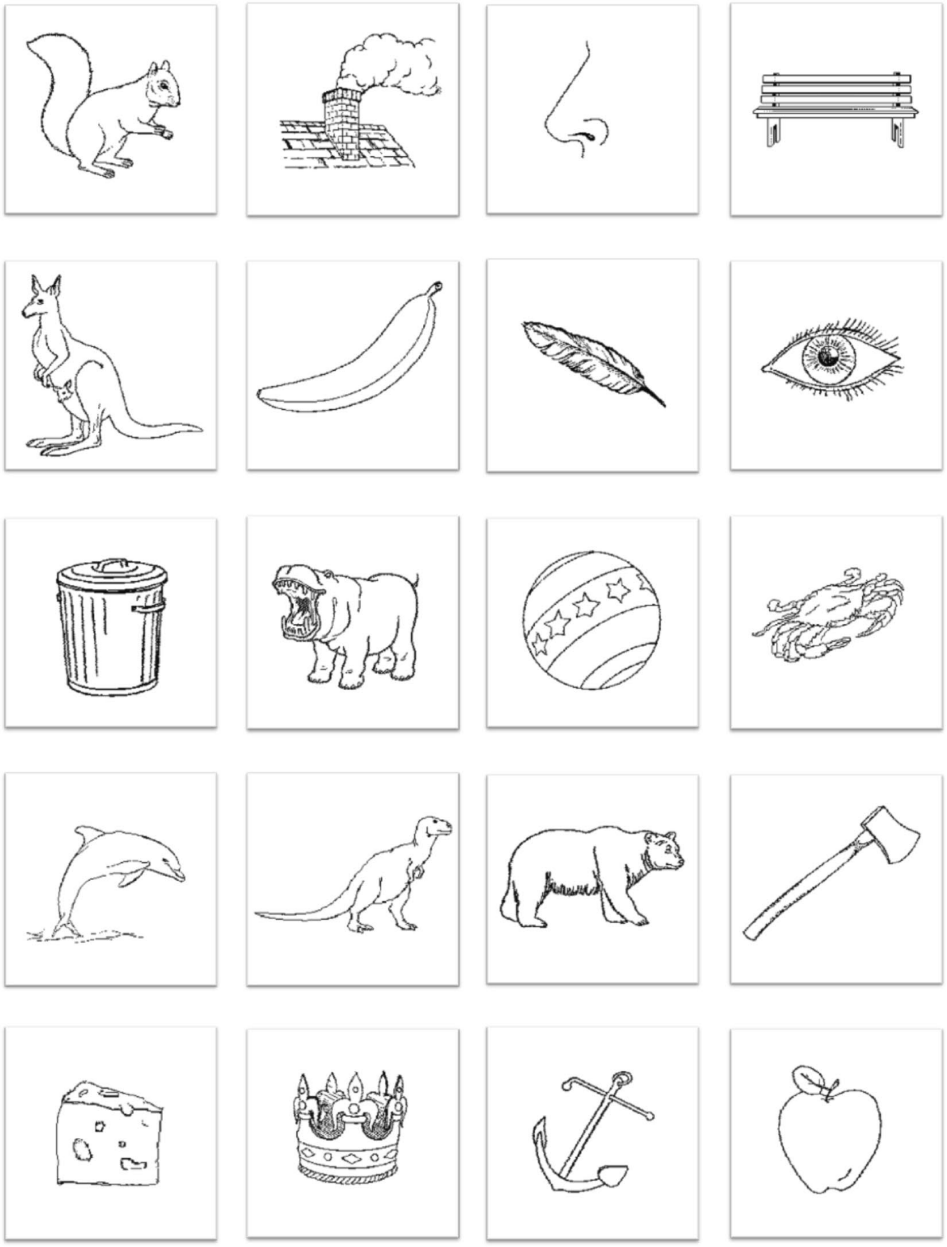
typical examples of the presented images. All images can be found in Alario and Ferrand database^26^.

***Task 1: Picture naming***. The naming task of this second dataset contains 40 unrecognizable scrambled objects on top of 80 meaningful pictures taken from the Alario and Ferrand database^25^ (see Fig. 3 for typical examples of the presented images). Scrambled pictures were generated from the Alario and Ferrand database by mixing drawings lines and participants were instructed to say nothing when viewing them. Pictures were displayed on a screen as black drawings on a white background. Pictures were selected to get a high name agreement (avg = 96.86%, min = 86%; max = 100%), see Table 3 for image’s parameters of dataset 2.

**Table 3:** Psycholinguistic parameters controlled to get a homogeneous dataset 2.

	Average	Min	Max
Name agreement (%)	96,86	86	100
Image agreement (average)	3,65	1,23	4,87
Age of acquisition (average)	2,21	1,12	3,65
Number of letters	6,42	3	10
Number of phonemes	4,74	2	8
Number of syllables	1,92	1	4
Oral frequency	55,68	0	892
Written frequency	36,48	0,03	476,69

We also controlled our data set to have as many semantical representatives at each end of some principal components continua whose spatial distribution on the surface of the brain has been demonstrated^27^ (mobile/non-mobile: 40 vs. 40; animacy/non-animacy: 46 vs 34; social/non-social: 30 vs. 50; civilization/nature: 41 vs. 39; animal/not-animal: 41 vs. 39; biological/non-biological 48 vs. 32). Ones could also check these dimensionality effects against controlled semantical information that are supposed not to produce any distinct spatial clusters as for example: portable vs. not portable (44 vs. 36). Some of these categories are adjustable depending on cultures.

The E-Prime 2.0 software (Psychology Software Tools, Pittsburgh, PA) was used to display the pictures. The typical trial starts with a fixation cross that lasts 1000 *ms*, then the image is shown during 2500 *ms* followed by a blank screen for 1000 or 2000 *ms* (randomly selected). The picture triggers are recorded when the image disappears from the screen (offset and not the onset as for dataset1). To get the onsets of a picture display, ones have to subtract 2500 ms. The time between the picture onset and the beginning of vocalization recorded by the system was considered as naming latencies. The voice onset times were then analyzed using Praat software^25^.

***Task 2: Auditory naming***. The participants were asked to name 80 different sounds from the NESSTI database^28^ and displayed through headphones. The duration of each sound is 1 second, reaction time between onset and oral response are calculated also using Praat v5.3.13^25^. All participant’s responses on played sounds are registered with the reaction time. Participants were asked to wait until the end of the sound to give their answer. This means that for this task in particular, voice onset time does not inform us on the rapidity of the cognitive process. Indeed, sound identification could have started and even ended during listening.

Sounds were selected according to a criterion of 50% or more of correct identification as mentioned in the study 1 of the NESSTI^28^. Like for the picture of the object naming task, we also controlled our data set to have as many semantical representatives at each end of some principal components continua whose spatial distribution on the surface of the brain has been demonstrated^27^ (mobile/non-mobile: 45 vs 35; animacy/non-animacy: 39 vs 41; social/non-social: 27 vs. 53; civilization/nature: 45 vs. 35; animal/not-animal: 39 vs.41; biological/non-biological 39 vs. 41). Ones could also check these dimensionality effects against controlled semantical information that are supposed not to produce any distinct spatial clusters as for example: smaller or bigger than a pigeon (41 vs. 39). Again, most of these semantic criteria can be adjusted according to cultures.

***Task 3: Working memory***. This task is the last of the session for each participant. 80 pictures were displayed of which 40 have already been shown in the naming task. New pictures and already seen pictures randomly appeared on the screen and participants have to indicate if they have seen them before by pressing a button or not. The 40 new images used in this task were controlled to match on average the psycholinguistic parameters of the 40 already seen images.

## Data Records

**Fig. 4:**
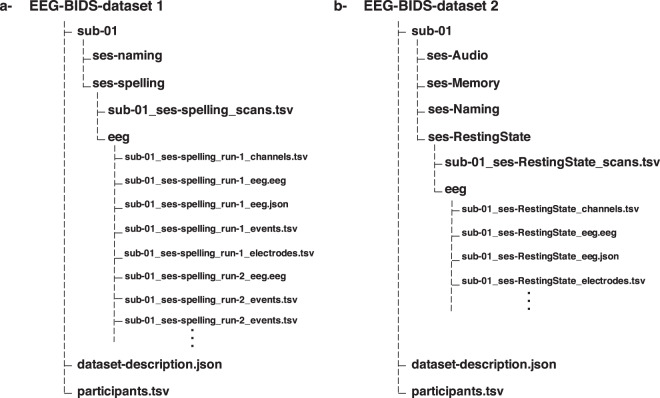
Exemplary EEG-BIDS for one participant in dataset 1 (**a**) and dataset 2 (**b**).

All the data are available in BIDS format^29,30^, and uploaded separately to OpenNeuro site (https://openneuro.org). The main folder of Dataset 1 (available at: 10.18112/openneuro.ds003420.v1.0.2)^31^ contains 23 folders, one for each participant, and two files: i) “data-description.json” that describes the dataset and contains information about where and when the data are registered and ii) “participants.tsv” that contains information about the participants such as sex, age and education level. Each participant’s folder contains two subfolders for the naming and spelling tasks, the two tasks folders contain the EEG data, channels, events, etc … for two sessions 1 and 2 (See Fig. 4). The same folder’s structure applies for the Dataset 2 (available at: 10.18112/openneuro.ds003421.v1.0.2)^32^ with 20 participants folders and four tasks folders for each participant (naming, audio, memory and resting state), each task folder contains the EEG data, events, etc.

## Technical Validation

### Preprocessing/data quality

**Fig. 5:**
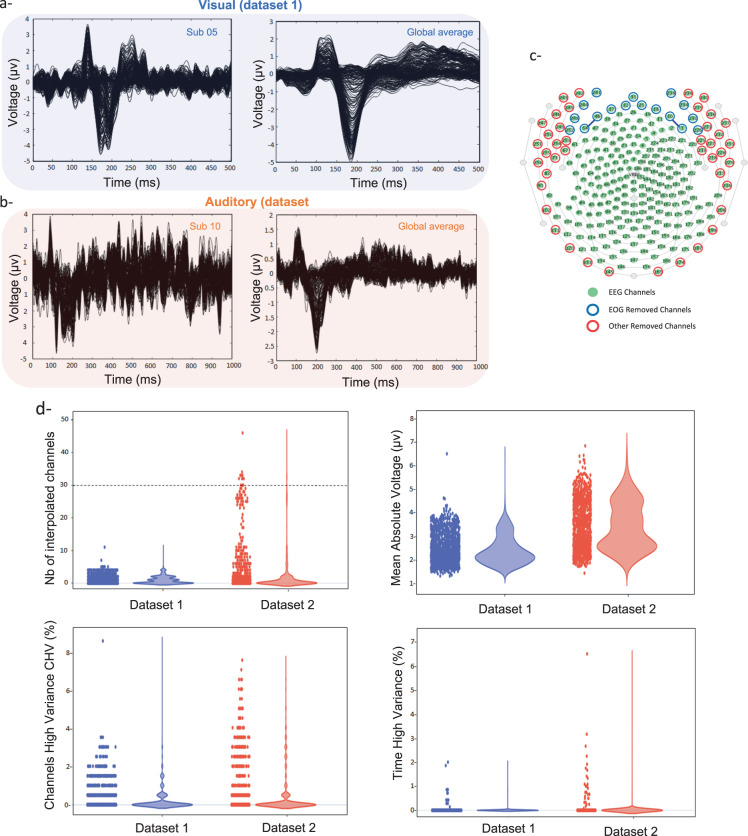
Preprocessing and data quality. (**a**) Average response over trials in the picture naming task (session 1 of dataset 1), left: sub-05, right: all participants. (**b**) Average response over trials in the auditory task of dataset 2, left: sub-10, right: all participants. (**c**) Sensor layout of the hydrocel geodesic system (256 channels, EGI) with the removed channels (blue and red) when performing preprocessing by Automagic. (**d**) Quality metrics across all participants and trials in dataset 1 and dataset 2, (top/left): number of interpolated channels per epoch, (top/right): mean absolute voltage per epoch, (bottom/left): percentage of channels for which the standard deviation across time exceeds 15 uV, (bottom/right): percentage of time points where the standard deviation across channels exceeds 15 uV.

First, we provide an overview qualitative and quantitative evaluation of the EEG signals quality of both datasets. Results are illustrated in Fig. 5. A typical example of the event related potentials (ERPs) for a given participant (averaged over trials) is illustrated in Fig. 5a,b (left) for both visual (from dataset 1) and auditory (from dataset 2), respectively. The global average ERPs (over trials and participants) are presented in Fig. 5a,b (right). The channels used to perform the electrooculography (EOG) regression and the removed channels (from Automagic) are presented in Fig. 5c. In total, 195 EEG channels (over 256 channels from the EGI system) were kept. Figure 5d provides four quantitative metrics used by Automagic as criteria to ensure the quality of the preprocessed signals. First, the number of interpolated channels is presented in Fig. 5d (up/left) showing that only few channels were interpolated (lower than the 15% of the channels).

The figure shows that more channels were interpolated in dataset 2 compared to dataset 1 and that some trials can be removed (above the yellow line denoting the 15% threshold). All the other metrics show very low values below the threshold set by Automagic used to accept/reject trials. See https://github.com/methlabUZH/automagic for more technical details about these metrics.

### Brain dynamics analysis

Several analyses can be done on this data such as time, frequency, time-frequency or connectivity-based analysis, among others. Here we provide an example of the analysis that can be done using these datasets to explore the dynamics of brain activity at scalp, source and network level. To do so, we use only open-source tools, in order to ease the reproducibility of these examples. A tool for automatic preprocessing will be used called Automagic https://github.com/methlabUZH/automagic, and another tool for the automatic segmentation (into microstates) from EEGLAB https://archive.compute.dtu.dk/files/public/users/atpo/Microstate. Results are visualized using either EEGLAB for voltage topography and BrainNet viewer https://www.nitrc.org/projects/bnv/ for the networks.

#### Dataset 1

**Fig. 6:**
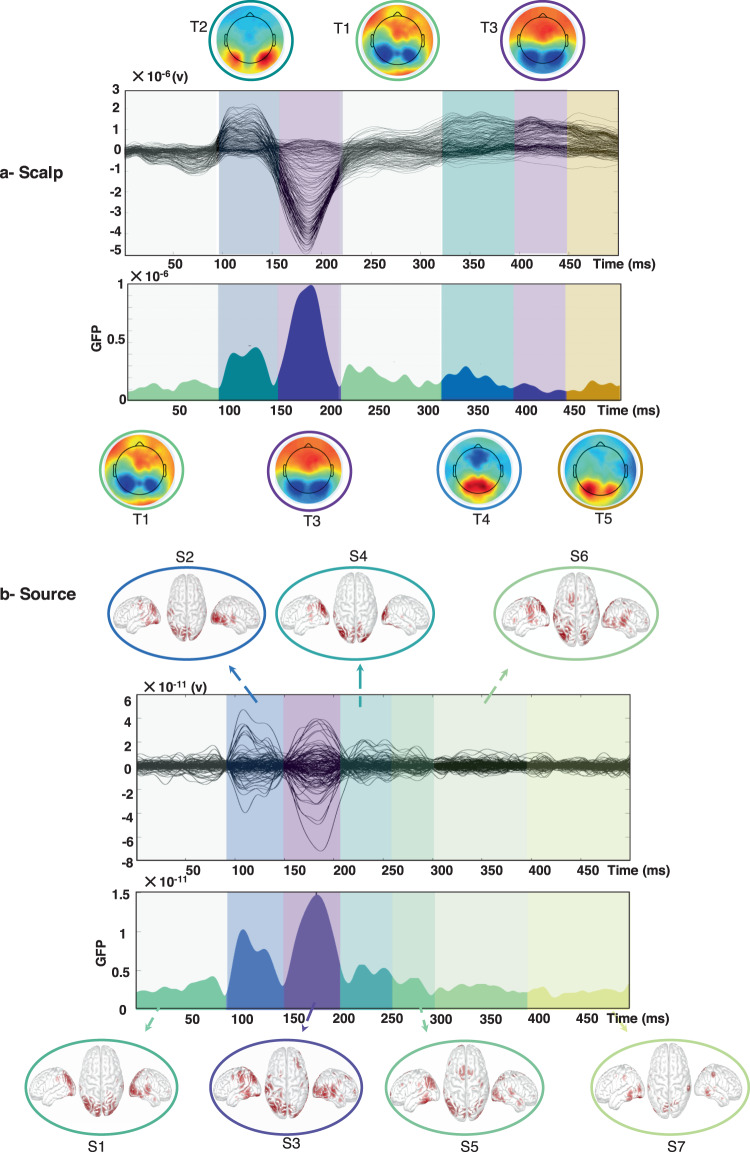
Results of the microstate segmentation algorithm applied on the ERP of picture naming task (**a**, **b**) at the source level, using the eeglab microstate toolbox^35^.

Validation of dataset 1 is performed on the picture naming task (described in section 2.1.1) at both scalp and cortical level. First, data are preprocessed using Automagic Matlab toolbox^33^. The preprocessed data are then uploaded to EEGLAB toolbox^34^, and the event response potential (ERP) is calculated over all 23 participants and trials. The ERP was segmented using a toolbox called “*Microstate eeglab toolbox*”^35^. The segmentation was done using the *K-means modified* algorithm and results showed five topography states (Fig. 6a).

The first state (T1) starts from 1 to 94 ms, the second state (T2) from 94 to 154 ms, the third state (T3) from 154 to 227 ms, the fourth state (T4) from 325 to 374 ms and the fifth state (T5) from 443 to 500 ms. Globally the results show a transition from the occipito-temporal lobe (T2, visual processing) to frontal lobe (T3) with a repetition of this spatial pattern between T4 and the second appearance of T3. Noteworthy, both these sequences are preceded by the T1 pattern that seems to correspond to a fronto-medial activation.

Second, the EEGs data and MRI template were coregistered using Brainstorm toolbox^36^ and Destrieux atlas used to parcellate the cortical surface onto 148 regions^37^. The source time series are reconstructed using the weighted minimum norm estimated (wMNE) method available in Brainstorm. The averaged regional time series over all participants is then segmented using the *k*-means modified algorithm integrated *in eeglab microstate toolbox*. The automatic segmentation using the same tool as for scalp showed seven states (Fig. 6b): the first state (S1) from 1 to 94 ms, the second state (S2) from 94 to 143 ms, the third state (S3) from 143 to 204 ms, the fourth state (S4) from 204 to 250 ms, the fifth state (S5) from 250 to 297 ms, the sixth state (S6) from 297 to 380 ms and the seventh state (S7) from 380 to 500 ms. Again, the results show a transition from the occipito-temporal lobe (S1-S2, visual processing) to fronto-central region (S3) and a repetition of this pattern between S4 and S5.

Reactivation of the occipital part at S7 overlap with the two topographic maps (second T3 and T5) of the previous analysis. Despite the different number of states in both cases, results show globally similar spatiotemporal activations with frontal and occipital activities and very precise time cuts especially for the first three topographies and source’s maps.

**Fig. 7:**
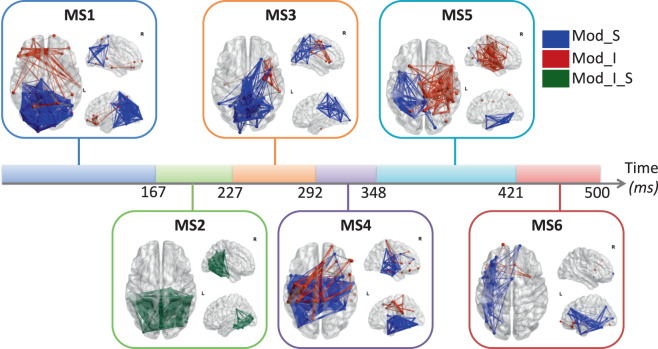
The sequential modular states of the EEG picture naming task obtained using the modularity-based segmentation algorithm^40^, where Mod_S represent a segregation module, Mod_I represents an integration module and Mod_I_S represents both integration and segregation module.

Third, the reconstructed regional time series were filtered in gamma band (30–45 Hz) and the functional connectivity were computed using phase looking value (PLV)^38^, using EEGNET toolbox^39^. To explore how brain networks reshape over time, we detected the time-varying dynamic modular states using a recently proposed and validated algorithm^40^. Other algorithms can be used also such as source separation or clustering approaches. Briefly, this algorithm attempts to extract the main modular structures (known as modular states) that fluctuate repetitively across time. Results presented in Fig. 7 showed six modularity states.

In Fig. 7, we show the modules that exhibit the highest segregation (more communication across modules, called Mod_S) and the highest integration (more communication within the module, called Mod_I) at each time window. Figure shows a transition from segregated/integrated occipito-temporal connections (MS2) during visual processing and recognition to segregated fronto-central functional connections (MS4). The transition from MS2 to MS4 adds information about communication between brain areas that is not available in the two previous analyses. Indeed, correlation in the gamma band between brain areas have been interpreted as the “binding” phenomenon (see^41^ or multidisciplinary references) or more extensively; to the matching of memory content and stimulus-related information and the use of^42^. However, users of this method should keep in mind that it is only in the gamma range and that it is hardly comparable to the previous results which use different characteristics of the broad signal.

The three level results (scalp, sources and network) are complementary and researchers can for sure run other types of analysis. Here we wanted to show a concert analysis on how to see the dynamics of brain activity at sub-second time scale. The three types of analysis can be easily reproduced using the tools described above. Globally, the three approaches show the implication of the visual cortex during this task where participants were asked to name ‘visual’ stimuli. All three methods can be used to detect limits of cognitive processes, but cognition can’t be reduced to a global mixture of signal nor to a specific frequency range. That is why the network method and the topographical method converge quite well on MS2 and T3 while the rises and falls of the amplitude of the sources makes it difficult to operate this division except when signal amplitude is at stake (transition S1 = >S2; T1 = >T2 at 94 ms).

#### Dataset 2

**Fig. 8:**
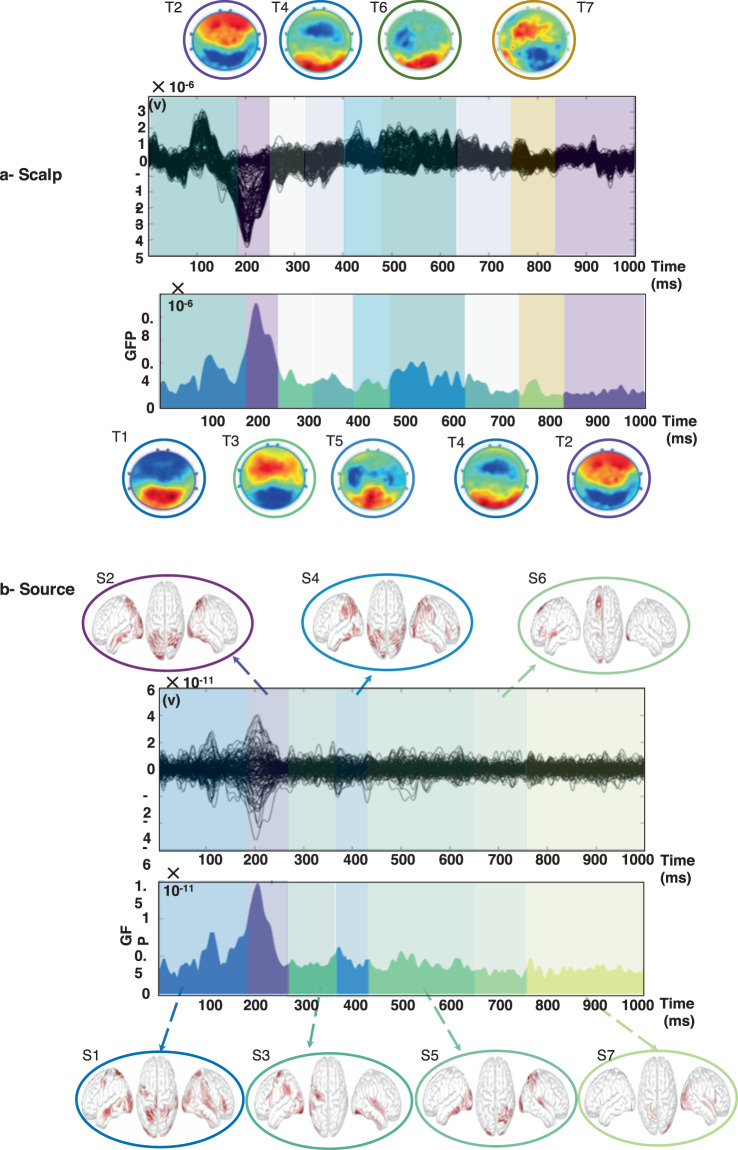
Results of the microstate segmentation algorithm applied on the ERP (**a**) the sources (**b**) using the EEGLAB microstate toolbox^35^.

**Fig. 9:**
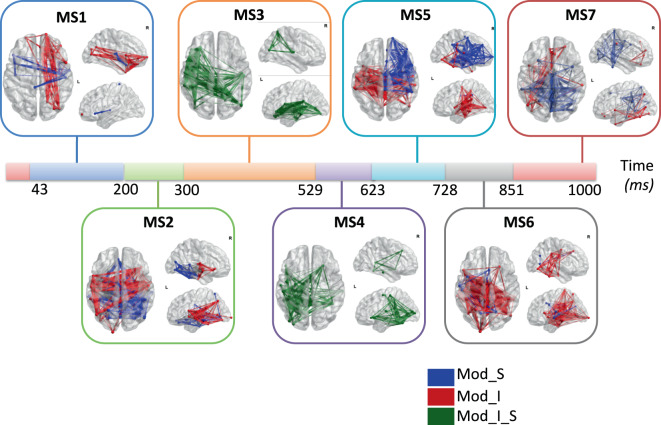
The sequential modular states of the EEG auditory task obtained using the modularity-based segmentation algorithm^40^, where Mod_S1 and Mod_S2 represent a segregation module, Mod_I represents an integration module and Mod_I_S represents both integration and segregation module.

Here we conducted the same analysis, as for the visual stimuli, on the auditory naming task. Participants were asked to name the objects they listen to. Scalp and source -level analyses are summarized in Fig. 8 and the source-space connectivity-based analysis (in the beta band) is illustrated in Fig. 9.

Figure 8 shows that at both scalp and source level, an activation in the parietal and temporal cortex can be observed (T2 and S2). But the most striking result of this temporal activity is represented by MS2 which is shifted of 30 ms in time. Again, the time limits of the naming process match well for its first steps between the topographical and source methodologies. Results showed also that the occipital cortex can be also involved (with less activation, in T4 and S4). As for the picture naming task, we observe here the same repetitive shift between sensorial areas dedicated to stimuli (temporo-parietal) and frontal areas involved in planification and task processing. This is well illustrated in the source domain by the sequence S2-S3 and again S4-S6 with S3 and S6 carrying the clusters in the frontal areas while S2 and S4 show exclusively temporo-parietal sources. Finally, Fig. 9 shows mainly implications of the parieto-temporal connections at the moment of processing/recognizing the auditory stimulus (MS2) then a network with temporo-frontal connections is observed (MS5) followed by networks that involves the central cortex (MS6 and MS7).

Like in the picture naming task where this period lasted only 58 ms; the auditory task analyzed with the network method reveals a step in the cognitive process where networks are neither segregated or integrated but fully merged from 300 ms to 623 ms. The interesting point is that here; we observe the beta activity which is known to reflect alertness instead of specific processing like learning or retrieving from memory that are supported or translated by an increase of gamma activity. This beta network (MS3-MS4) that lasts 323 ms could correspond to the time during which the participant is trying to recognize the heard sounds. and, consequently, the participant is perfectly on alert.

This second analysis on the dataset 2 shows again that all three methods are complementary. It also shows that results are still consistent despite less participants and less correct responses (auditory identification led to fewer correct responses). Finally, it shows that the network method can bring different results given the frequency band at stake in the analysis.

## Usage Notes

Some of the most common software packages for analyzing these data are freely available, and include FieldTrip (http://fieldtrip.fcdonders.nl/), MNE (http://martinos.org/mne/), Brainstorm (http://neuroimage.usc.edu/brainstorm), EEGLAB (http://sccn.ucsd.edu/eeglab/), and EEGNET(https://sites.google.com/site/eegnetworks/), Automagic (https://github.com/methlabUZH/automagic). The code used to identify the modularity states is available at https://github.com/librteam/Modularity_algorithm_NN.

This dataset has multiple potential uses for cognitive neuroscience and for methodological development in EEG analysis, such as:

1. The analysis of the brain dynamics at sub-second time scale in different conditions such as comparing picture naming vs. spelling.
2. The validation of several parameters involved in the methods used to estimate cortical brain networks (called EEG source connectivity) through scalp EEG, such as the open question of optimal number of channels. Using 256 channels, this gives the possibility to sub-sample the channel density and test other EEG channel density such as 128, 64, 32 and 19.
3. The comparison between the dynamic reconfiguration of functional brain networks for different modality such as visual vs. auditory and the comparison of task-related and task-free networks. The possibility to predict performance during tasks from resting data can be also investigated for researchers working in the domain of brain decoding using machine learning approaches for instance.
